# Digital cognition plus plasma p‐Tau217 and Aβ42/40 powerfully predict Alzheimer's progression

**DOI:** 10.1002/dad2.70281

**Published:** 2026-02-28

**Authors:** Ahmet Begde, Yi Yang, Vanessa Raymont, James B. Rowe, Dean Palejev, Dishaa Sinha, Matthew Bennett, Rachel Brohier, Hannah Rome‐Hall, Ivan Koychev, for the Alzheimer's Disease Neuroimaging Initiative

**Affiliations:** ^1^ Department of Psychiatry University of Oxford Oxford UK; ^2^ Department of Clinical Neurosciences University of Cambridge Cambridge UK; ^3^ Medical Research Council Cognition and Brain Sciences Unit Cambridge UK; ^4^ Cambridge University Hospitals NHS Foundation Trust Cambridge UK; ^5^ Institute of Mathematics and Informatics Bulgarian Academy of Sciences Sofia Bulgaria; ^6^ Faculty of Mathematics and Informatics Sofia University Sofia Bulgaria; ^7^ Department of Brain Sciences Imperial College London London UK

**Keywords:** amyloid, blood biomarkers, digital cognitive assessment, phosphorylated tau, positron emission tomography, p‐tau217, tau

## Abstract

**INTRODUCTION:**

Alzheimer's disease is characterized by amyloid‐β (Aβ) and tau accumulation. Identifying individuals with rapid proteinopathy progression is crucial for timely intervention and trial enrichment.

**METHODS:**

We analyzed longitudinal data from 456 Alzheimer's Disease Neuroimaging Initiative (ADNI) participants, including 375 with digital cognitive test scores. Amyloid and tau accumulation rates were estimated from positron emission tomography (PET) imaging using linear mixed‐effects models. Participants were classified as fast or slow accumulators via Gaussian modeling. Predictors of accumulation and clinical conversion were assessed with logistic and Cox regression models, incorporating demographics, cognitive measures and plasma biomarkers.

**RESULTS:**

Plasma p‐tau217 and Aβ42/Aβ40 predicted rapid accumulation and conversion, with p‐tau217 the strongest marker (odds ratio [OR] up to 6.6). Baseline digital cognitive measures contributed significantly to the prediction, achieving comparable or superior predictive accuracy to traditional cognitive tests (area under the curve [AUC] up to 0.92; C‐index 0.82).

**DISCUSSION:**

Plasma p‐tau217 and Aβ42/Aβ40 emerged as robust predictors of the progression of disease pathology, supported by cognitive measures.

## Highlights

- Plasma p‐tau217 and amyloid‐β (Aβ) 42/Aβ40 ratio powerfully predict rapid amyloid and tau accumulation patterns, enabling identification of fast progressors
- Digital cognitive tests achieve comparable or superior predictive accuracy to traditional neuropsychological tests when combined with plasma biomarkers
- A minimal multimodal panel of plasma p‐tau217, Aβ42/Aβ40 ratio, and digital cognitive assessment robustly predicts clinical conversion from normal cognition to mild cognitive impairment (MCI)/dementia, supporting scalable screening approaches

## 1 BACKGROUND

Alzheimer's disease (AD) is characterized by the accumulation of amyloid‐beta plaques and tau tangles in the brain.^1^ The rate of this accumulation varies markedly across individuals. Some people show fast pathological changes, while others remain stable for years.^2^ These pathological features may begin to accumulate years before noticeable symptoms appear, resulting in a long preclinical period during which brain damage progresses asymptomatically.^3^, ^4^

The potential for early diagnosis of AD extends to the detection of individuals at risk for clinical conversion.^5^, ^6^, ^7^ So far, risk stratification has focused primarily on risk factor composites (e.g., Cardiovascular Risk Factors, Aging and Dementia Study [CAIDE] score, apolipoprotein E [*APOE*] genetics) with the addition of cross‐sectional neurodegeneration biomarkers that indicate preclinical pathophysiology. From longitudinal risk monitoring cohorts, it has emerged that the speed of proteinopathy accumulation carries qualitatively distinct information on dementia risk. Identifying biologically at‐risk individuals through their rate of accumulation of abnormal proteins, in addition to genetic and clinical risk factors, could more effectively identify those at risk, enabling individualized drug and non‐drug interventions.

We have shown that episodic memory performance and *APOE* ε4 status are linked to positron emission tomography/cerebrospinal fluid (PET/CSF) amyloid and tau accumulation patterns in cognitively healthy aging populations.^8^ We showed that there is an inflection point in rapid accumulation that takes place in the early 50s and late 50s for APOE ε4 carriers and non‐carriers, respectively. Recent progress in blood biomarkers (BBMs), especially plasma amyloid‐beta ratios and phosphorylated tau (p‐tau), offers a biomarker sampling method that would more feasibly allow the serial testing required to identify rapid abnormal protein accumulators relative to cerebrospinal fluid tests and neuroimaging scans.^9^ Such plasma biomarkers accurately detect AD pathological change.^10^, ^11^

Cognitive assessments (both digital and paper‐and‐pencil) can also detect subtle changes in cognitive skills even before clinical symptoms appear.^12^, ^13^ Digital tools could provide advantages over paper‐based tests by reducing assessor bias, enhancing reliability through high‐density testing, and enabling more accurate, longitudinal assessment of cognitive changes.^14^, ^15^ However, their accuracy compared to traditional tests remains unclear.^16^, ^17^

BBMs, combined with digital cognitive assessments, could be a promising approach for detecting preclinical or prodromal AD in real‐world settings.^14^ Together, they can be a robust tool for identifying people at risk of future AD, enabling timely intervention and better patient outcomes. Davos Alzheimer's Collaborative launched a program to develop an effective early detection framework by combining an innovative digital screening assessment and a BBM test in primary care settings.^18^ However, further research is needed to validate their effectiveness, cost‐efficiency, and acceptance in broader populations.

Beyond early detection, understanding differences in accumulation trajectories can improve risk stratification and enable more effective intervention strategies. Identifying those on an aggressive AD progression remains challenging, yet such individuals are likely to benefit most from targeted interventions. Identifying a minimal marker combination with high predictive ability is crucial for cost‐effective screening.^19^

The primary aim of this study was to identify a minimal set of multimodal biomarkers that effectively predicts rapid versus slow pathological accumulation of amyloid and tau. We used longitudinal data from the Alzheimer's Disease Neuroimaging Initiative (ADNI) cohort, to test the hypothesis that plasma biomarkers—particularly p‐tau217 and amyloid‐β (Aβ) 42/Aβ40 ratio—are the most robust predictors, and that adding cognitive measures provides incremental predictive value. A secondary aim was to compare the predictive utility of digital versus traditional cognitive assessments when combined with plasma biomarkers.

## 2 METHODS

Data used in the preparation of this article were obtained from the ADNI database (adni.loni.usc.edu). The ADNI was launched in 2003 as a public‐private partnership, led by Principal Investigator Michael W. Weiner, MD. The primary goal of ADNI has been to test whether serial magnetic resonance imaging (MRI), positron emission tomography (PET), other biological markers, and clinical and neuropsychological assessment can be combined to measure the progression of mild cognitive impairment (MCI) and early AD. The present study focused on participants with available longitudinal PET imaging, BBMs and cognitive tests, resulting in a matched cohort of 456 individuals. The datasets used in this study are provided in Table S1.

When limiting the sample to those who completed the digital cognitive assessments, the number of eligible participants was lower. Specifically, 375 individuals had both digital cognitive data and longitudinal PET scans, compared to 456 with available traditional ADNI composite cognitive scores. In addition to demographics (age, sex and education), medical history data, including self‐reported comorbidities (cardiovascular/neurologic/psychiatric disease), apolipoprotein E (*APOE*) carrier status and family history of dementia, were used in this study. *APOE* ε4 carriers were identified as those individuals with one or more *APOE* ε4 alleles. ADNI was approved by local institutional review boards (IRBs) and obtained informed consent.

**RESEARCH IN CONTEXT**

1. **Systematic review**: We reviewed the literature using traditional online databases. Recent evidence showed that the speed of amyloid‐β (Aβ) and tau aggregation is a critical indicator of clinical progression. This study builds upon previous work, which demonstrates that individuals with rapidly accelerating pathology might be at high risk for cognitive decline.
2. **Interpretation**: Our findings demonstrate that plasma phosphorylated tau217 (p‐tau217) and the Aβ42/Aβ40 ratio are the most robust and accessible predictors of this aggressive trajectory, outperforming established risk factors like apolipoprotein E (APOE) ε4. Furthermore, we show that digital cognitive measures provide predictive value comparable to traditional neuropsychological tests when combined with blood biomarkers.
3. **Future directions**: Our study emphasizes that prediction based on established risk factors alone is modest, highlighting the critical need for dynamic, pathology‐specific biomarkers. Future work should validate this minimal, multimodal model (plasma p‐tau217 + Aβ42/40 + digital cognition) in diverse, real‐world settings to enable cost‐effective, scalable screening and monitoring.

### 2.1 Cognitive tests

Four ADNI composite scores were used to assess cognitive domains: ADNI‐Mem (memory), ADNI‐EF (executive function), ADNI‐Lan (language), and ADNI‐VS (visuospatial). These composite scores were calculated using cognitive test scores (Mini‐Mental State Examination [MMSE], Rey's Auditory Verbal Learning Test [RAVLT], Alzheimer's Disease Assessment Scale – Cognitive Subscale [ADAS‐cog], etc.) from the cognitive battery administered in ADNI, which could provide more robust and sensitive cognitive measures than individual tests alone.^20^, ^21^, ^22^

Digital cognitive assessment was performed using the Cogstate Brief Battery (CBB) consisting of four tests: (i) Detection Test measures psychomotor function through simple reaction time (RT) in milliseconds; (ii) Identification Test assesses visual attention via choice RT; (iii) One Card Learning Task evaluates visual recognition using accuracy scores; and (iv) One Back Task also measures working memory using RT. Digital cognitive assessments were performed either in a clinic (supervised) or at home remotely (unsupervised).

### 2.2 Plasma biomarkers

Four blood biomarkers were used in this study: Aβ42/Aβ40 ratio, p‐tau217, glial fibrillary acidic protein (GFAP), and neurofilament light chain (NfL). The ADNI biorepository contains BBMs data from multiple assay platforms that have been previously compared for their predictive capabilities (Schindler et al., 2024). Based on the results of comparative studies and the highest data availability through the ADNI portal, the Fujirebio Lumipulse assays for Aβ42/Aβ40 ratio and p‐tau217, and the Quanterix Neurology 4‐Plex E assays for GFAP and NfL were included for our analyses.

### 2.3 PET imaging biomarkers

Amyloid PET imaging was performed at each ADNI center using standardized acquisition protocols for both [18F]florbetaben (FBB; 90–110 min) and [18F]florbetapir (FBP; 50–60 min) tracers. The overall standardized uptake value ratio (SUVR) was calculated using cortical summary regions of interest (ROIs). Aβ burden was quantified in centiloid units using ADNI PET core conversion equations for florbetapir and florbetaben SUVR. The Centiloid calculation method was previously described.^23^

Tau PET imaging was conducted using the tracer [18F]‐flortaucipir (AV‐1451). Tracer uptake was quantified using 12 regional SUVRs, with the inferior cerebellum as the reference region across all cortical ROI.^24^ To summarize regional uptake, we employed a composite temporal meta‐ROI, based on established protocols.^24^ Tau PET positivity was initially assessed using a previously proposed SUVR threshold of 1.34 within the temporal meta‐ROI.^25^

### 2.4 Data analysis

Participants with and without digital cognitive assessment data were compared using Mann–Whitney *U* tests for continuous variables and Fisher's exact tests for categorical variables. Effect sizes were expressed as Cohen's d and Cramer's V, respectively. Of 456 participants, 25 lacked follow‐up amyloid PET data, leaving 431 for amyloid analyses. Missing values (<10%) were cross‐checked and filled from the ADNI merged datasets when possible. For variables with <10% missing data, remaining missing values after cross‐checking were left as missing and retained in the dataset without further imputation. Variables with >10% missingness were excluded.

Linear mixed effects (LME) models were fitted separately for longitudinal amyloid and tau PET data to estimate individual rates of pathological accumulation. The models included random intercepts and slopes to account for within‐subject correlation over time, adjusting for age at baseline, sex, education, and *APOE* ε4 status. For amyloid PET, the outcome was centiloid units; for tau PET, the global SUVR score was used. Model fit was evaluated using log‐likelihood values, and fixed‐effect estimates were reported with standard errors, *p*‐values, and 95% confidence intervals.

Participant‐specific slopes for amyloid and tau accumulation were extracted from the LME models. Individual amyloid and tau PET trajectories over time are shown in Figure S1, illustrating accumulation patterns across groups. Gaussian Mixture Models (GMM) were then applied to these slope estimates to classify individuals into fast (FA) and slow (SA) accumulation groups (see Figure S2). The threshold for classifying fast accumulators was set at the mean plus two standard deviations of the lower distribution component, as done in the previous ADNI study.^25^ Silhouette scores, ranging from 0 to 1, were used to evaluate model quality, with higher scores indicating better‐defined and more distinct clusters. To formally assess whether the distributions of slopes were unimodal, Hartigan's Dip Test was performed, with a high *p*‐value indicating no significant deviation from unimodality.

Following that, block‐based logistic regression models were developed to identify predictors of FA versus SA patterns for amyloid and tau, and of clinical conversion (cognitively normal to MCI/dementia, or MCI to dementia). Predictors were entered in sequential blocks, including demographics, medical history and genetics, cognitive function (traditional paper‐based vs. digital), and plasma biomarkers. Backward elimination was used within blocks to refine the models. All models controlled for visit type to ensure digital cognitive performance was estimated independently of the administration setting. Model performance was evaluated using the area under the receiver operating characteristic curve (AUC), Kappa statistics, sensitivity, specificity, and McNemar's test for classification agreement.

Cox proportional hazards regression models were also used to assess predictors of time to clinical conversion, incorporating the same predictor blocks as in the logistic models. Hazard ratios with 95% confidence intervals and *p*‐values were reported for significant predictors.

All statistical analyses were performed using R (version 4.4.3) and Python. Specifically, GMM for clustering analyses was conducted in Python, while regression and mixed‐effects models were implemented in R using relevant packages, including lme4 for LME models and glm for logistic regression. All statistical tests were two‐sided, and significance was set at *p* < 0.05.

## 3 RESULTS

The full cohort included 456 participants, of whom 375 had digital cognitive assessment data, and 81 did not. Compared to participants without digital assessment data, those with digital data were significantly younger (*p* = 0.035), had more years of education (*p* = 0.012), and demonstrated higher MMSE scores (*p* < 0.001). No significant differences were observed in sex, APOE ε4 carrier status, family history of dementia, or cardiovascular, neurologic, or psychiatric history (all *p* > 0.05; Table 1).

**TABLE 1:** Baseline demographic and clinical characteristics of the full cohort and digital cognitive assessment subgroup.

Characteristic (baseline)	Full cohort (*n* = 456)	With digital cognitive assessment data (*n* = 375)	Without digital cognitive assessment data (*n* = 81)	*p*‐Value	Effect size
Age, years (mean ± SD)	73.1 ± 7.5	72.8 ± 7.1	74.4 ± 8.8	0.035	0.213 (d)
Male sex, n (%)	222 (49.7%)	181 (49.3%)	41 (50.6%)	0.715	0.012 (V)
Education, years (mean ± SD)	16.4 ± 2.5	16.5 ± 2.5	15.8 ± 2.5	0.012	0.296 (d)
*APOE* ε4 carriers, n (%)	195 (42.8%)	156 (41.6%)	39 (48.1%)	0.322	0.045 (V)
Family history dementia, n (%)	156 (34.2%)	132 (35.2%)	24 (29.6%)	0.368	0.039 (V)
Cardiovascular history, n (%)	297 (65.1%)	242 (64.5%)	55 (67.9%)	0.609	0.021 (V)
Neurologic history, n (%)	128 (28.1%)	105 (28%)	23 (28.4%)	0.999	<0.001 (V)
Psychiatric history, n (%)	147 (32.2%)	121 (32.3%)	26 (32.1%)	0.999	<0.001 (V)
MMSE score (mean ± SD)	28.2 ± 2.5	28.6 ± 1.7	26.2 ± 3.9	<0.001	1.087 (d)

*Note*: Effect size: Cohen's d for continuous variables; Cramer's V for categorical variables.

Abbreviations: *APOE* ε4, apolipoprotein E epsilon 4 allele; MMSE, Mini‐Mental State Examination; SD, standard deviation.

### 3.1 Linear mixed effects model for longitudinal tau and amyloid PET

Longitudinal PET data were analyzed using LME modeling to quantify individual tau and amyloid accumulation rates (see Table S2).

#### 3.1.1 Amyloid model (centiloid)

**FIGURE 1:**
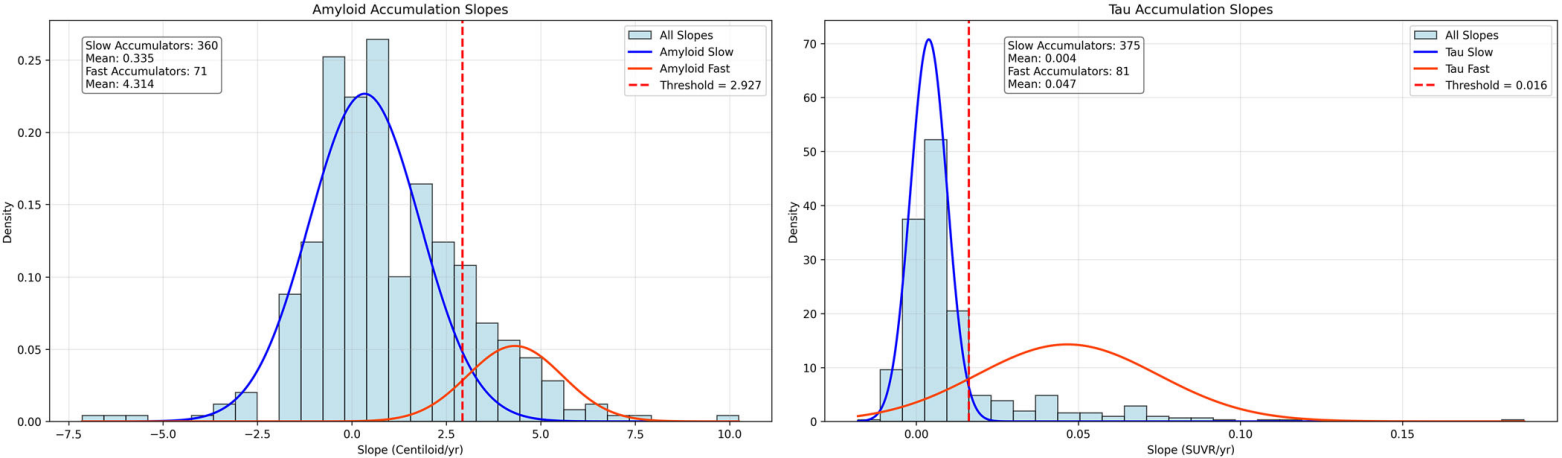
Gaussian mixture model clustering across amyloid and tau accumulation subgroups.

The GMM outcome for clustering the individuals as SAs or FAs is shown below (see Figure 1). The distribution of the annual rates of amyloid accumulation (centiloid) can be explained by two components (lower: mean  =  0.37, SD  =  1.27; higher: mean  =  1.95, SD  =  2.60). Individuals with an annual rate of accumulation ≥ lower mean + (2 × lower SD) ≈2.93 were clustered as FAs. Silhouette score of 0.55 suggests moderate separation between the clusters. Classifications are moderately confident (average max prob = 0.76, Akaike Information Criterion [AIC]: 1821.74, Bayesian Information Criterion [BIC]: 1842.07). FAs (*n* = 71) have significantly higher mean slope (4.31, SD = 1.26) compared to the SAs (*n* = 360, mean = 0.33, SD = 1.47, *p* = <0.001), confirming that the threshold effectively distinguishes between the two groups. Characteristics of FAs and SAs were compared in Table 2. Participants classified as fast amyloid or tau accumulators exhibited higher baseline PET scores than SAs (see Table 2).

**TABLE 2:** Demographic and genetic characteristics of fast and slow amyloid and tau accumulators.

Variable	Amyloid fast accumulators (*n* = 71)	Amyloid slow accumulators (*n* = 360)	*p*‐value	Tau fast accumulators (*n* = 81)	Tau slow accumulators (*n* = 375)	*p*‐Value
Baseline Amyloid/Tau (centiloid/SUVR, Mean ± SD)	65.79 ± 43.35	23.45 ± 38.89	**<0.001**	1.52 ± 0.33	1.14 ± 0.07	**<0.001**
Slope (Mean ± SD)	4.31 ± 1.26	0.33 ± 1.47	**<0.01**	0.047 ± 0.03	0.004 ± 0.01	**<0.01**
Age (Mean ± SD)	74.6 ± 6.2	72.2 ± 7.4	**0.01**	73.2 ± 7.6	73.1 ± 7.4	0.89
Education (Mean ± SD)	16.5 ± 2.4	16.4 ± 2.5	0.95	16 ± 2.3	16.5 ± 2.6	0.13
*APOE* ε4 Carriers	36 (50.7%)	145 (40.3%)	0.13	54 (66.7%)	141 (37.6%)	**<0.01**
Male Participants	34 (47.9%)	171 (47.5%)	0.99	38 (46.9%)	171 (49.1%)	0.81

*Note*: Bold values indicate statistical significance (p < 0.05).

Abbreviations: *APOE* ε4, apolipoprotein E epsilon 4 allele; SD, standard deviation; SUVR, standardized uptake value ratio.

#### 3.1.2 Tau model (global SUVR)

GMM using the annual rates of tau accumulation (global SUVR) showed two groups: a lower‐rate group (mean = 0.004, SD = 0.006) and a higher‐rate group (mean = 0.043, SD = 0.030). Individuals with an annual rate of accumulation ≥ lower mean + (2 × lower SD) ≈ 0.016 were clustered as FAs. Silhouette score of 0.77 indicates well‐separated clusters. Individuals are classified into one cluster with very high confidence (max class prob = 0.96, AIC: −2787.29, BIC: −2766.68). FAs (*n* = 81) have significantly higher mean slope (0.047, SD = 0.028) compared to the SAs (*n* = 375, mean = 0.004, SD = 0.006, *p* = <0.001), showing that the threshold effectively distinguishes between the two groups. Hartigan's dip test indicated no significant deviation from unimodality for either marker (Amyloid: dip = 0.0149, *p* = 0.7912; Tau: dip = 0.0087, *p* = 0.9972), suggesting that while subgroups can be defined for comparison, the overall slope distributions are statistically unimodal.

### 3.2 Logistic regression model

To minimize redundancy and multicollinearity, pairwise correlations were examined across candidate variables. Based on these results, a backward elimination modeling approach was adopted. Please see the correlation matrix (see Figure S3).

- **Model 1 – Baseline**: Demographics (age, sex, education)
- **Model 2 – Medical History**: Model 1 plus medical comorbidities (cardiovascular, neurological, and psychiatric history; family history of dementia, *APOE* ε4 status).
- **Model 3 – Cognitive Function (traditional or digital)**: Model 2 plus traditional neuropsychological assessments (ADNI composite scores for memory, executive function, language, and visuospatial abilities) or computerized cognitive performance measures (reaction times for working memory, detection, and identification tasks; learning accuracy).
- **Model 4 – Biomarkers**: Model 3 plus plasma biomarkers (Aβ42/40, p‐tau217, GFAP, and NfL).

#### 3.2.1 Predicting amyloid accumulator patterns

**FIGURE 2:**
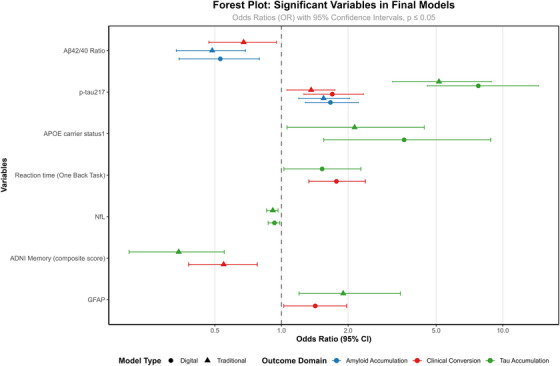
Forest plot of variables in the final model: Digital vs. traditional assessments.

**FIGURE 3:**
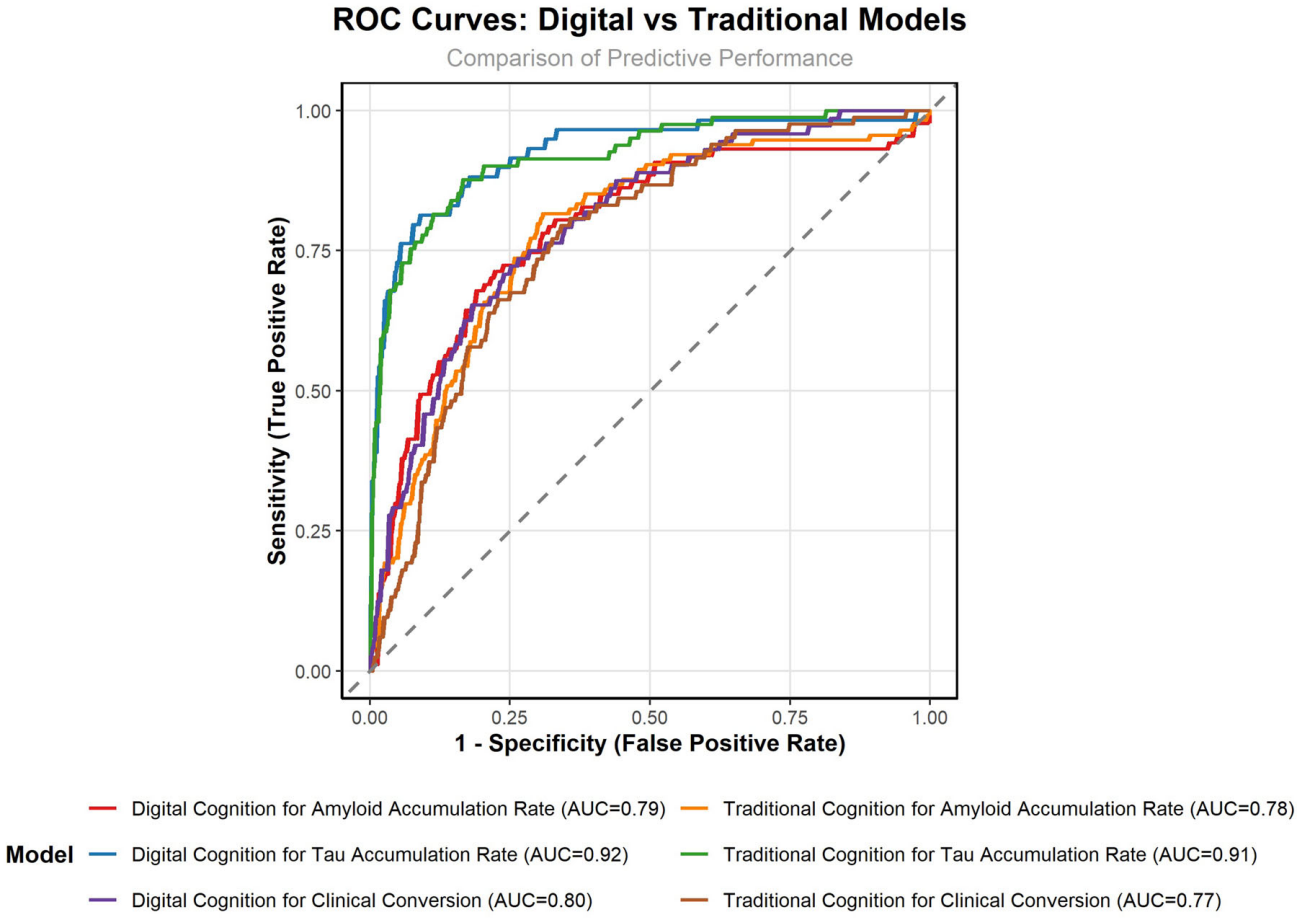
Receiver operating characteristic (ROC) curves comparing predictive performance of digital vs. traditional cognitive assessments.

The logistic regression models showed that plasma biomarkers (Aβ42/Aβ40 ratio and p‐tau217) were the only consistently significant variables in both final digital versus traditional models (see Figure 2/Table 3). *APOE* ε4 status showed statistical significance at Block 2 in both models (see Table S3). The ADNI Memory Test was significant only in the traditional model (Block 3), and all digital cognitive assessments were eliminated in the digital model. Both models showed very similar accuracy predicting performance (AUC 0.78 vs. 0.79, see Figure 3 and Table S4).

**TABLE 3:** Traditional vs. digital cognitive assessments in predicting amyloid accumulation, tau accumulation, and clinical conversion.

Domain	Variable	Odds/hazard ratio (traditional)	95% CI (traditional)	*p*‐Value (traditional)	Odds/hazard ratio (digital)	95% CI (digital)	*p*‐value (digital)
Amyloid accumulation	Plasma Aβ42/Aβ40	0.49	0.34–0.69	<0.001	0.53	0.35–0.79	0.002
	Plasma p‐tau217	1.55	1.20–2.03	0.001	1.66	1.28–2.23	<0.001
Tau accumulation	*APOE* ε4 Status	2.14	1.06–4.42	0.03	4.01	1.77–9.70	<0.001
	ADNI Memory Test	0.34	0.21–0.55	<0.001	–	–	–
	One Back RT	–	–	–	1.51	1.04–2.24	0.03
	Plasma p‐tau217	5.15	3.18–8.88	<0.001	6.39	3.91–11.36	<0.001
	Plasma GFAP	1.93	1.22–3.50	0.02	–	–	–
	Plasma NfL	0.91	0.86–0.96	0.02	0.85	0.72–0.97	0.03
Clinical conversion (logistic)	ADNI Memory Score	0.55	0.37–0.78	<0.001	–	–	–
	One Back RT	–	–	–	1.84	1.38–2.50	<0.001
	p‐tau217	1.37	1.06–1.75	0.01	1.63	1.22–2.23	0.001
	Aβ42/Aβ40	0.68	0.47–0.95	0.03	–	–	–
	GFAP	–	–	–	1.43	1.03–1.98	0.03
Clinical conversion (Cox)	*APOE* ε4 Status	–	–	–	1.70	1.03–2.80	0.03
	ADNI Memory Score	0.47	0.34–0.64	<0.001	–	–	–
	One Back RT	–	–	–	1.69	1.38–2.06	<0.001
	p‐tau217	1.38	1.16–1.63	<0.001	1.91	1.62–2.26	<0.001
	Aβ42/Aβ40	0.68	0.50–0.93	0.014	–	–	–

Abbreviations: Aβ42/Aβ40, amyloid‐beta 42 to 40 ratio; ADNI, Alzheimer's Disease Neuroimaging Initiative; *APOE*, apolipoprotein E; CI, confidence interval; GFAP, glial fibrillary acidic protein; HR, hazard ratio; NfL, neurofilament light chain; OR, odds ratio; p‐tau217, phosphorylated tau 2.

#### 3.2.2 Predicting tau accumulator patterns

The same models were used to identify the minimal set of variables that best predict the binary tau accumulation pattern (fast vs. slow). Across both models, *APOE* ε4 status and plasma biomarkers (p‐tau217, GFAP, and NfL) were consistently significant predictors in the final model (see Figure 2). In the traditional cognitive model, the ADNI Memory composite was a significant predictor. In contrast, in the digital cognitive model, two Cogstate tasks (One Back and One Card Learning) entered the model, but only One Back RT remained statistically significant in the final model.

The digital model showed slightly higher AUC (0.92 vs. 0.91) while the traditional model had slightly higher specificity and Kappa statistic (see Figure 3/Table S4).

To further investigate early disease mechanisms, we conducted a subgroup analysis restricted to participants who were amyloid‐negative at baseline (*n* = 229 for amyloid; *n* = 232 for tau, see Table S5). Most individuals in this cohort were classified as SAs. Specifically, only 10% (*n* = 23) were fast amyloid accumulators, and 2.6% (*n* = 6) were fast tau accumulators.

In the amyloid accumulation model, psychiatric history was a significant predictor, alongside plasma p‐tau217 and Aβ42/40. Notably, the digital model proved more sensitive than the traditional model, identifying another reaction time task (detection) (OR = 2.29, *p* < 0.001) as a key predictor where traditional tests failed to show significance. For tau accumulation, plasma p‐tau217 remained the strongest predictor.

Although only 2.6% were fast tau accumulators, plasma p‐tau217 remained a highly significant predictor. The digital reaction time score slightly contributed to the digital model, demonstrating that digital tools can identify tau progression even in the absence of baseline amyloid pathology (see Figure S4). Importantly, APOE ε4 status was not a significant predictor of tau accumulation in this amyloid‐negative group (*p* = 0.171), suggesting its role is primarily linked to established amyloid pathology rather than early tau pathology in this specific subgroup (see Table S6).

#### 3.2.3 Predicting clinical conversion (normal to MCI/dementia)

These models aim to identify the minimal set of variables that best predict binary clinical conversion classification patterns (converter vs. non‐converter, from cognitively normal (CN) to MCI/dementia or from MCI to dementia), and to compare the predictive utility of digital cognitive assessments and traditional paper‐based cognitive assessments. Among participants with digital cognitive data (*n* = 375), baseline classification showed 251 CN, 120 MCI, and 4 dementia, with 72 converting during follow‐up. The traditional cognitive assessment group (*n* = 456) included 287 CN, 144 MCI, and 25 dementia at baseline, with 83 converting. Dementia cases were excluded from conversion models.

In the digital cognitive model, One Back RT was identified as a strong predictors, with two other plasma biomarkers (p‐tau217 and GFAP) in the final model (Table 3). The traditional model also retained the ADNI Memory cognitive composite score with plasma biomarker (p‐tau 217 and Aβ42/Aβ40 Ratio, see Figure 2). The digital model showed higher AUC and Kappa score (see Table S4).

### 3.3 Cox regression models

To provide a more comprehensive understanding of clinical conversion risk, Cox regression (time‐to‐event) analysis (which accounts for the timing of conversion) was also performed to understand how early different predictors affect progression risk. Both models used Cox proportional hazards regression with block‐wise backward elimination, incorporating demographics, medical history, *APOE* ε4 status, cognitive measures (digital vs. traditional), and plasma biomarkers.

Model performance was high across both approaches, with C‐indices of 0.81 (traditional) and 0.82 (digital), indicating strong discriminative ability. Time‐dependent receiver operating characteristics (ROC) areas under the curve (AUCs) were similarly robust (0.77 vs. 0.79; see Table S4). Overall, results support the complementary value of plasma biomarkers and cognitive measures—whether digital or traditional—in predicting clinical progression. Digital and Traditional cognitive tests provided very similar predictive performance.

## 4 DISCUSSION

Our study demonstrates that a minimal set of biomarkers‐plasma p‐tau217, Aβ42/Aβ40 ratio, and either a traditional memory composite or a digital working memory measure‐robustly predicts amyloid/tau accumulation and clinical conversion. Our results show that digital working‐memory measures provide comparable or superior predictive utility to traditional tests when paired with plasma biomarkers. This finding supports scalable, low‐cost approaches for early detection and risk stratification in AD. By integrating multimodal data, we offer a data‐driven framework for precision risk stratification and clinical trial enrichment.

The classification of individuals into FA and SA revealed considerable variation in both tau and amyloid accumulation rates, consistent with previous studies demonstrating heterogeneous AD progression.^26^, ^27^ The role of age and *APOE* ε4 was significant in predicting faster accumulation. Amyloid accumulation was more age‐related, occurring gradually and significantly earlier in *APOE* ε4 carriers, but with age being the stronger determinant. Tau accumulation, on the other hand, demonstrated a stronger direct link to *APOE* ε4 compared to age, particularly in accelerating earlier onset and progression. This is consistent with previous studies that have reported that *APOE* ε4 carriers exhibit faster accumulation.^8^, ^28^, ^29^ A study of the accelerated failure time model showed that *APOE* ε4 carriers had an accelerated pathological amyloid accumulation by 6.1 years (5.0–7.2) and accelerated tau accumulation by 2.6 years (1.2–4.1) compared to non‐carriers.^30^ Age was also a significant covariate in amyloid accumulation and clinical conversion models. However, the interaction between age and *APOE* genotype needs to be further investigated, as recent studies have suggested that *APOE* ε4 may accelerate tau accumulation at lower amyloid levels in older carriers.^31^

Plasma p‐tau217 was identified as the strongest and most consistent biomarker across predictive models for both pathological accumulation and clinical conversion. This aligns with previous evidence reporting p‐tau217 as a highly specific marker of AD pathology.^10^, ^11^, ^32^ Higher p‐tau217 plasma levels were associated with higher odds ratios for being a fast tau accumulator (OR up to 6.64) and increased hazard ratios (HR) for clinical conversion (HR up to 1.96). These results align with previous findings reported in earlier single‐cohort or multicohort studies.^9^, ^33^, ^34^ Moreover, some models showed that combining plasma p‐tau217 with the Aβ42/Aβ40 ratio would enhance classification performance, supporting recent findings that dual‐target biomarker panels can effectively capture both amyloid pathology and tau‐related neurodegeneration.^35^

Interestingly, while GFAP and NfL contributed more prominently to tau accumulation prediction than amyloid markers, NfL showed an unexpected negative association. A previous paper reported that NfL correlates more with clinical progression rather than early pathological changes.^36^ NfL may also reflect broader neurodegenerative processes not directly tied to tau pathology, which could highlight the complexity of biomarker interrelationships.^37^, ^38^

This study also reported the head‐to‐head comparison of traditional (ADNI composite) and digital (Cogstate Brief Battery) cognitive assessments in predicting pathological accumulation and clinical progression. Neither digital nor traditional cognitive scores contributed additional predictive value for amyloid accumulation beyond plasma biomarkers. This could be due to amyloid's subtle preclinical pathological changes before cognitive deficits, which are better predicted by plasma biomarkers due to their sensitivity. However, for tau accumulation and clinical conversion, digital metrics—particularly a working memory RT test (One Back)—offered comparable or superior predictive ability to traditional memory scores. This is supported by recent evidence showing that a memory digital assessment distinguished between amyloid PET‐positive patients better than standard MoCA (AUC 0.77 vs. 0.74).^39^ Another recent study also showed that combining a brief self‐administered digital cognitive test with a blood biomarker achieved up to 90% accuracy in detecting AD in primary care, significantly outperforming standard cognitive tests, physician assessments, and biomarker testing alone.^40^

Similarly, our prior work in the EPIC Norfolk cohort found that a digital visual processing reaction time test showed comparable predictive ability for future dementia diagnosis compared to paper‐based memory and verbal learning tests, but demonstrated higher sensitivity to dementia risk factors.^41^ As demonstrated by Davos Alzheimer's Collaborative and other real‐world screening initiatives,^18^, ^42^ digital assessments can be sensitive, scalable tools for longitudinal cognitive monitoring—particularly when paired with plasma biomarkers. Our results support recent calls to streamline diagnostic pathways using combinations of BBMs and cognitive tools in primary care settings.^14^, ^43^

The retention of different cognitive predictors across models, episodic memory versus reaction time, is likely due to the complexity of AD pathology. Traditional memory tests might show structural loss, while reaction time might measure early changes in processing speed. This difference may also be influenced by our statistical approach, as backward elimination can select different predictors when variables are correlated, and sample characteristics vary. Identifying fast tau and amyloid accumulators is critical for prognosis and trial stratification. To focus on fast progressors rather than include slow progressors would make trials more efficient, and potentially improve the risk‐benefit ratio for treatments. With growing investment in anti‐amyloid therapies (e.g., lecanemab, donanemab), there is an urgent need for practical screening tools to identify candidates before severe neurodegeneration.^44^

Limitations of this study include limited diversity in the ADNI cohort (predominantly White individuals, highly educated), which may reduce generalizability. The mean follow‐up duration (2.1–2.4 years) is relatively short and may underestimate long‐term disease trajectories. Digital cognitive performance could be affected by the familiarity with technology which may limit generalizability, highlighting the need for validation in younger and more diverse populations. Additionally, digital cognitive assessments were administered under supervised clinical conditions, so results need validation in real‐world, unsupervised settings.

Future research should validate these models in diverse cohorts and use machine learning to capture complex interactions (e.g., age × APOE ε4). Importantly, pragmatic trials should assess the cost‐effectiveness and acceptability of implementing plasma + digital screening strategies in routine practice.^45^

It will also be valuable to explore the predictive performance in younger populations, as ADNI's mean cohort age was over 70. This could help determine how early and reliably one can identify faster accumulators.^46^

In conclusion, our study demonstrates that a minimal multimodal panel, plasma p‐tau217 and Aβ42/Aβ40 ratio combined with cognitive assessments, effectively classifies and predicts amyloid/tau accumulation patterns and risk stratification of clinical conversion to AD. We showed that non‐invasive digital tools are scalable and sensitive, performing comparably to traditional neuropsychological batteries for progression forecasting, supporting their use for widespread screening and remote monitoring. With continued validation, multimodal strategies could significantly advance our ability to predict, prevent and prepare AD progression to improve patient outcomes.

## CONFLICT OF INTEREST STATEMENT

A.B., Y.Y., D.P., M.B., H.R.‐H., R.B., and D.S. declare no competing interests. J.B.R. has provided consultancy to Alector, Asceneuron, Astronautx, Alzheimer's Research UK, Astex, Aviado Bio, Booster Therapeutics, ClinicalInk, Curasen Therapeutics, Cumulus Neuro, Eisai, Ferrer, SV Health, Prevail, Vesper Bio and UCB; and received research grants from AstraZeneca, Lilly, GSK and Janssen as part of the Dementias Platform UK and is Chief Scientific Advisor to Alzheimer's Research UK. I.K. is a paid medical advisor for digital healthcare (Five Lives SAS, Lola Speaks) and biotechnology companies (Prima Mente, Oxford Brain Diagnostics), which have a focus on neurodegeneration. I.K. has received non‐promotional speaker fees from Novo Nordisk and Eisai, and for advisory work for Johnson and Johnson, Zylorion and Novo Nordisk. He is in receipt of a grant for an investigator‐initiated study from Novo Nordisk. Author disclosures are available in the supporting information.

## ETHICS STATEMENT

Each site involved in data collection for the ADNI received local IRB approval. Written informed consent was obtained from each enrolled participant.

## Supporting information



Supporting Information

Supporting Information

## Data Availability

The results presented in this study are based on data from ADNI. ADNI data are publicly available through the ADNI Portal (https://ida.loni.usc.edu or https://adni.loni.usc.edu). The analysis code will be made available upon request through a GitHub repository.
