# Supplementary material for: Digital cognition plus plasma p‐Tau217 and Aβ42/40 powerfully predict Alzheimer's progression
Source: Alzheimers Dement (Amst). 2026 Feb 28;18(1):e70281. doi: 10.1002/dad2.70281 (PMC12949451; doi:10.1002/dad2.70281)

**Supplementary materials**

**Tables**

1. **Supplementary Table 1. Data Sources and Variables Used in the Analysis**

| Data Category | Variables Used | ADNI File |
| --- | --- | --- |
| Amyloid PET | Sumamry SUVR, Centiloids | UC Berkeley - Amyloid PET 6mm Res analysis [ADNI1,GO,2,3,4] |
| Tau PET | Meta Temporal SUVR Regional SUVRs | UC Berkeley - Tau PET PVC 6mm Res analysis [ADNI2,3,4] |
| Blood Biomarkers | Fuji-Plasma Aβ42/Aβ40, p-tau217 and QX-GFAP, NfL (Quanterix) | UPENN - Plasma Biomarkers (AB42, AB40, ptau217, NfL, GFAP) measured by Fujirebio & Quanterix [ADNI1,GO,2,3,4] |
| Digital Cognitive | Cogstate battery (Reaction times, accuracy) | Cogstate Battery Results [ADNI2,3] |
| Neuropsychological | ADNI composite scores | UW - Neuropsych Summary Scores [ADNI1,GO,2,3] |
| Demographics | Age, sex, education, APOE4 | Medical History [ADNI1,GO,2], Vital Signs [ADNI1,GO,2,3,4], ADNIMERGE - Key ADNI tables merged into one table - Packages for R [ADNI1,GO,2], Subject Demographics [ADNI1,GO,2,3,4] |
| Clinical Diagnosis | Diagnosis | Diagnostic Summary [ADNI1,GO,2,3,4] |

1. **Supplementary Table 2. Linear Mixed-Effects Model Results for Amyloid and Tau Accumulation**

Longitudinal PET data were analysed using LME modelling adjusted by age, education, *APOE* ε4 genotype, and sex to quantify individual tau and amyloid accumulation rates. An LME model was used to assess the effects of time, age, education, *APOE* ε4 genotype, and sex on amyloid burden. The model included 1,222 observations from 431 participants, with a mean follow-up time of 2.4 years. The log-likelihood of the model was –5359.26, and the estimated population-level slope of amyloid accumulation was 0.9902 centiloids per year, with a slope ranging from –7.14 to 10.24. The model included random intercepts and slopes for time by participant. Time from baseline (β = 0.99, p = .002) and age at scan (β = 1.20, p < .001) were significantly associated with increased amyloid burden. In contrast, higher education was associated with significantly lower amyloid burden (β = -2.55, p = .001). *APOE* ε4 allele count had a strong effect on amyloid burden (β = 27.78, p < .001). Sex was not determined to be significant.

The LME model for Tau burden included 1,278 observations from 456 participants, with a mean follow-up time of 2.1 years. The log-likelihood of the model was 920.40, and the estimated population-level slope of tau accumulation was 0.012 SUVR per year. The model was adjusted for age, education, *APOE* ε4 status, and sex. The model included random intercepts and slopes for time by participant. Time from baseline (β = 0.01, p < .001) and *APOE* ε4 status (β = 0.04, p = .008) were significantly associated with increased tau burden. Age, sex and education were not determined to be significant (p > .05).

| **Variable** | **Amyloid Burden Coefficient (β)** | **SE (Amyloid)** | **p-value (Amyloid)** | **95% CI (Amyloid)** | **Tau Burden Coefficient (β)** | **SE (Tau)** | **p-value (Tau)** | **95% CI (Tau)** |
| --- | --- | --- | --- | --- | --- | --- | --- | --- |
| Time from baseline | 0.99 | 0.32 | 0.002 | 0.35 – 1.63 | 0.011 | 0.002 | <0.001 | 0.008 – 0.015 |
| Age at baseline | 1.20 | 0.26 | <0.001 | 0.69 – 1.72 | 0.001 | 0.001 | 0.97 | -0.002 – 0.002 |
| Sex (Male=1) | 2.60 | 3.80 | 0.49 | –4.84 – 10.05 | 0.022 | 0.017 | 0.18 | –0.010 – 0.055 |
| Education (years) | –2.55 | 0.76 | 0.001 | –4.05 – –1.06 | –0.004 | 0.003 | 0.20 | –0.011 – 0.002 |
| APOE status (Neg=0) | 27.78 | 3.11 | <0.001 | 21.66 – 33.89 | 0.037 | 0.014 | 0.008 | 0.010 – 0.065 |

1. **Supplementary Table 3. Stepwise Logistic Regression Models for Predicting Pathological Accumulation and Clinical Conversion**

Both traditional and digital cox models identified age, *APOE* ε4 carrier status, and cardiovascular co-morbidity as significant predictors of progression starting from Block 2. As cognitive variables were introduced, the ADNI Memory Score remained highly significant in the traditional model (Blocks 3 and 4), while One-Back Reaction Time was a strong predictor in the digital model. Importantly, plasma p-tau217 was significant in both models in the final block, with a higher hazard ratio in the digital model (HR = 1.96 vs. 1.38), suggesting a more pronounced association with progression risk when digital cognitive metrics are used. The Aβ42/Aβ40 ratio was only significant in the traditional model.

| **Domain** | **Block** | **Variable** | **Odds/Hazard Ratio (Traditional)** | **95% CI (Traditional)** | **p-value (Traditional)** | **Odds/Hazard Ratio (Digital)** | **95% CI (Digital)** | **p-value (Digital)** |
| --- | --- | --- | --- | --- | --- | --- | --- | --- |
| **Amyloid Accumulation** | Block 2 | APOE Status | 2.10 | 1.35–3.28 | 0.001 | 2.23 | 1.35–3.68 | 0.002 |
|  | Block 3 | APOE Status | 1.98 | 1.26–3.11 | 0.003 | 2.23 | 1.35–3.68 | 0.002 |
|  |  | ADNI Memory Test | 0.54 | 0.40–0.72 | <0.001 | — | — | — |
|  | Block 4 | Plasma Aβ42/Aβ40 | 0.49 | 0.34–0.69 | <0.001 | 0.53 | 0.35–0.79 | 0.002 |
|  |  | Plasma p-tau217 | 1.55 | 1.20–2.03 | 0.001 | 1.66 | 1.28–2.23 | <0.001 |
| **Tau Accumulation** | Block 2 | APOE4 Status | 3.59 | 2.16–6.09 | <0.001 | 3.59 | 2.16–6.09 | <0.001 |
|  | Block 3 | APOE4 Status | 2.96 | 1.69–5.31 | 0.001 | 3.51 | 2.09–6.05 | <0.001 |
|  |  | ADNI Memory Test | 0.21 | 0.14–0.31 | <0.001 | — | — | — |
|  |  | One-Back RT | — | — | — | 1.42 | 1.05–1.87 | 0.02 |
|  |  | One-Card Learning | — | — | — | 0.07 | 0.01–0.33 | 0.002 |
|  | Block 4 | *APOE* ε4 Status | 2.14 | 1.06–4.42 | .03 | 4.01 | 1.77–9.70 | <.001 |
|  |  | ADNI Memory Test | 0.34 | 0.21–0.55 | <0.001 | — | — | — |
|  |  | One-Back RT | — | — | — | 1.51 | 1.04–2.24 | .03 |
|  |  | Plasma p-tau217 | 5.15 | 3.18–8.88 | <0.001 | 6.39 | 3.91–11.36 | <.001 |
|  |  | Plasma GFAP | 1.93 | 1.22–3.50 | 0.02 | — | — | — |
|  |  | Plasma NfL | 0.91 | 0.86–0.96 | 0.02 | 0.85 | 0.72–0.97 | 0.03 |
| **Clinical Conversion (Logistic)** | Block 2 | Age | 1.05 | 1.01–1.09 | 0.01 | 1.05 | 1.01–1.09 | 0.009 |
|  |  | APOE4 Status | 1.73 | 1.06–2.84 | 0.02 | 1.87 | 1.10–3.21 | 0.02 |
|  | Block 3 | ADNI Memory Score | 0.42 | 0.30–0.58 | <0.001 | — | — | — |
|  |  | One-Back RT | — | — | — | 4.22 | 2.77–6.92 | <0.001 |
|  | Block 4 | ADNI Memory Score | 0.55 | 0.37–0.78 | <0.001 | — | — | — |
|  |  | One-Back RT | — | — | — | 1.84 | 1.38–2.50 | <.001 |
|  |  | p-tau217 | 1.37 | 1.06–1.75 | 0.01 | 1.63 | 1.22–2.23 | .001 |
|  |  | Aβ42/Aβ40 | 0.68 | 0.47–0.95 | 0.03 | — | — | — |
|  |  | GFAP | — | — | — | 1.43 | 1.03–1.98 | .03 |
| **Clinical Conversion (Cox)** | Block 1 | Age | 1.04 | 1.01–1.07 | 0.022 | 1.04 | 1.00–1.07 | 0.031 |
|  | Block 2 | Cardiovascular Co-morbidity | 1.75 | 1.02–2.99 | 0.040 | 1.49 | 1.02–2.01 | 0.047 |
|  |  | *APOE* ε4 Status | 2.07 | 1.31–3.25 | 0.001 | 2.03 | 1.24–3.30 | 0.001 |
|  | Block 3 | ADNI Memory Score | 0.34 | 0.26–0.46 | <0.001 | — | — | — |
|  |  | One-Back RT | — | — | — | 1.82 | 1.47–2.22 | <0.001 |
|  | Block 4 | *APOE* ε4 Status | — | — | — | 1.70 | 1.03–2.80 | .03 |
|  |  | ADNI Memory Score | 0.47 | 0.34–0.64 | <.001 | — | — | — |
|  |  | One Back RT | — | — | — | 1.69 | 1.38–2.06 | <.001 |
|  |  | p-tau217 | 1.38 | 1.16–1.63 | <.001 | 1.91 | 1.62–2.26 | <.001 |
|  |  | Aβ42/Aβ40 | 0.68 | 0.50–0.93 | .014 | — | — | — |

Aβ42/Aβ40 = Amyloid-beta 42 to 40 ratio; *APOE* = Apolipoprotein E; CI = Confidence Interval; GFAP = Glial Fibrillary Acidic Protein; HR = Hazard Ratio; NfL = Neurofilament Light Chain; OR = Odds Ratio; p-tau217 = Phosphorylated Tau 2

1. **Supplementary Table 4. Summary of Model Performance Metrics**

| **Domain** | **Metric** | **Traditional Model** | **Digital Model** |
| --- | --- | --- | --- |
| **Amyloid Accumulation** | Kappa | 0.24 | 0.22 |
|  | AUC (ROC) | 0.78 | 0.79 |
|  | Sensitivity | 94.6% | 96.3% |
|  | Specificity | 24.5% | 20.6% |
|  | McNemar’s Test p-value | <0.001 | <0.001 |
| **Tau Accumulation** | Kappa | 0.65 | 0.606 |
|  | AUC | 0.91 | 0.92 |
|  | Balanced Accuracy | 79% | 76.5% |
|  | Sensitivity | 97% | 97.3% |
|  | Specificity | 62% | 55.7% |
|  | McNemar’s Test p-value | 0.003 | <0.001 |
| **Clinical Conversion (Logistic)** | Kappa | 0.10 | 0.31 |
|  | AUC | 0.77 | 0.81 |
|  | Sensitivity | 97.2% | 96.3% |
|  | Specificity | 9.6% | 27.7% |
|  | McNemar’s Test p-value | <0.001 | <0.001 |
| **Clinical Conversion (Cox)** | C-index | 0.81 | 0.82 |
|  | Time-dependent AUC | 0.77 | 0.79 |

1. **Supplementary Table 5. Linear Mixed-Effects Model Results for Amyloid and Tau in Amyloid-Negative Subgroup**

An LME model was used to assess the effects of time, age, education, APOE ε4 genotype, and sex on amyloid burden. The updated model included 649 observations from 229 participants, with a mean follow-up of 2.8 years. The model included random intercepts and slopes for time by participant. In this updated analysis, time from baseline was significantly associated with increased amyloid burden (β = 0.552 centiloids per year, p < 0.001), and APOE ε4 allele count was also significantly associated with higher amyloid burden (β = 3.301, p = 0.008). Age at baseline (β = -0.071, p = 0.405), sex (β = -1.856, p = 0.119), and education (β = 0.027, p = 0.912) were not significantly associated with amyloid accumulation. Random effects indicated variability across participants in both baseline amyloid burden and slope over time.

The LME model for tau burden included 603 observations from 232 participants, with random intercepts and slopes for time by participant. In this updated model, time from baseline was not significantly associated with tau accumulation (β = 0.00007 SUVR per year, p = 0.949). Sex was significantly associated with tau burden, with males showing slightly lower tau (β = -0.022, p = 0.043), and education was also significantly associated, with higher education linked to slightly higher tau burden (β = 0.004, p = 0.047). Age at baseline (β = 0.001, p = 0.168) and APOE ε4 count (β = -0.016, p = 0.171) were not significantly associated with tau accumulation. Random effects indicated modest variability in baseline tau levels across participants.

| **Variable** | **Amyloid Coefficient (β)** | **SE (Amyloid)** | **p-value (Amyloid)** | **Tau Coefficient (β)** | **SE (Tau)** | **p-value (Tau)** |
| --- | --- | --- | --- | --- | --- | --- |
| **Time from baseline** | 0.552 | 0.141 | <0.001 | 0.00007 | 0.001 | 0.949 |
| **Age at baseline** | -0.071 | 0.085 | 0.405 | 0.001 | 0.001 | 0.168 |
| **Sex (Male = 1)** | -1.856 | 1.191 | 0.119 | -0.022 | 0.011 | 0.043 |
| **Education (years)** | 0.027 | 0.242 | 0.912 | 0.004 | 0.002 | 0.047 |
| **APOE ε4 count** | 3.301 | 1.243 | 0.008 | -0.016 | 0.011 | 0.171 |

Participant-specific amyloid accumulation slopes, extracted from the LME model, were analysed using a 2-component Gaussian Mixture Model. The GMM identified two distinct components: a lower accumulation group (Component 1) with a mean slope of 0.478 ± 0.495 Centiloids/year (weight = 0.761), and a higher accumulation group (Component 2) with a mean slope of 0.790 ± 1.244 Centiloids/year (weight = 0.239). A threshold for classification was set at the lower component mean plus two standard deviations (1.467 Centiloids/year).

Based on this threshold, participants were classified as fast accumulators (n = 23, 10.0%) or slow accumulators (n = 206, 90.0%). Fast accumulators had higher slopes and showed significantly lower baseline amyloid Centiloids (mean = -6.96) compared to slow accumulators (mean = -0.06; Mann–Whitney U test, p = 0.000).

A separate GMM analysis using a stricter slope threshold (0.0063 SUVR/year) identified fast tau accumulators (n = 6, 2.6%) and slow tau accumulators (n = 226, 97.4%). Fast tau accumulators had higher slopes (mean = 0.0140 SUVR/year vs. -0.0003 SUVR/year) but did not differ significantly from slow accumulators in age, education, APOE4 status, or sex.

1. **Supplementary Table 6. Logistic Regression Models for Predicting Accumulation in Amyloid-Negative Subgroup**

| **Domain** | **Variable** | **Odds Ratio (Traditional)** | **95% CI (Traditional)** | **p-value (Traditional)** | **Odds Ratio (Digital)** | **95% CI (Digital)** | **p-value (Digital)** |
| --- | --- | --- | --- | --- | --- | --- | --- |
| **Amyloid Accumulation** | Psychiatric History | 2.666 | 1.010–7.323 | .050 | 5.029 | 1.478–20.688 | .014 |
|  | Calculated Aβ42/Aβ40 | 0.405 | 0.247–0.636 | <.001 | 0.394 | 0.210–0.706 | .002 |
|  | Plasma p-tau217 | 1.500 | 0.994–2.100 | .026 | 1.614 | 1.109–2.426 | .010 |
|  | Detection Reaction Time | — | — | — | 2.290 | 1.517–3.617 | <.001 |
| **Tau Accumulation** | Detection Reaction Time | — | — | — | 5.697 | 1.494–69.178 | .049 |
|  | Plasma p-tau217 | 2.576 | 1.560–5.145 | .001 | 3.924 | 1.630–20.641 | .020 |
|  |  |  |  |  |  |  |  |

**Figures**

**1. Supplementary Figure 1. Individual amyloid and tau PET trajectories over time**


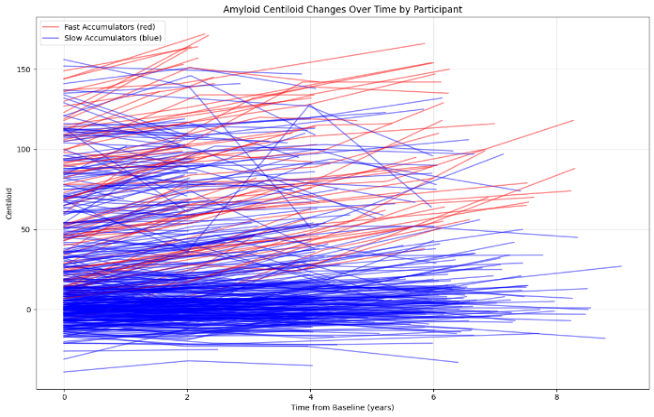

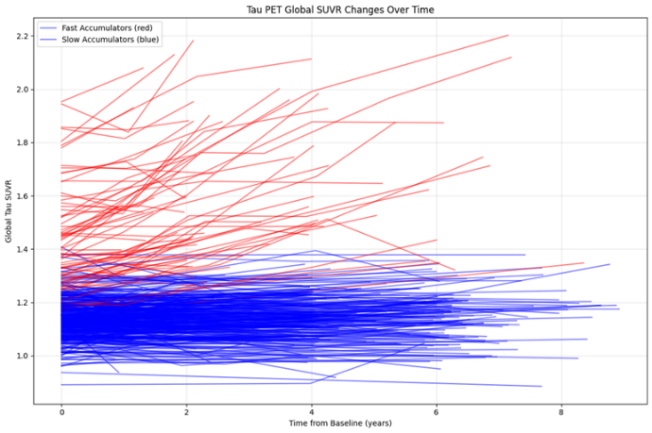


1. **Supplementary Figure 2. Gaussian Mixture Model components for classifying fast and slow accumulators**


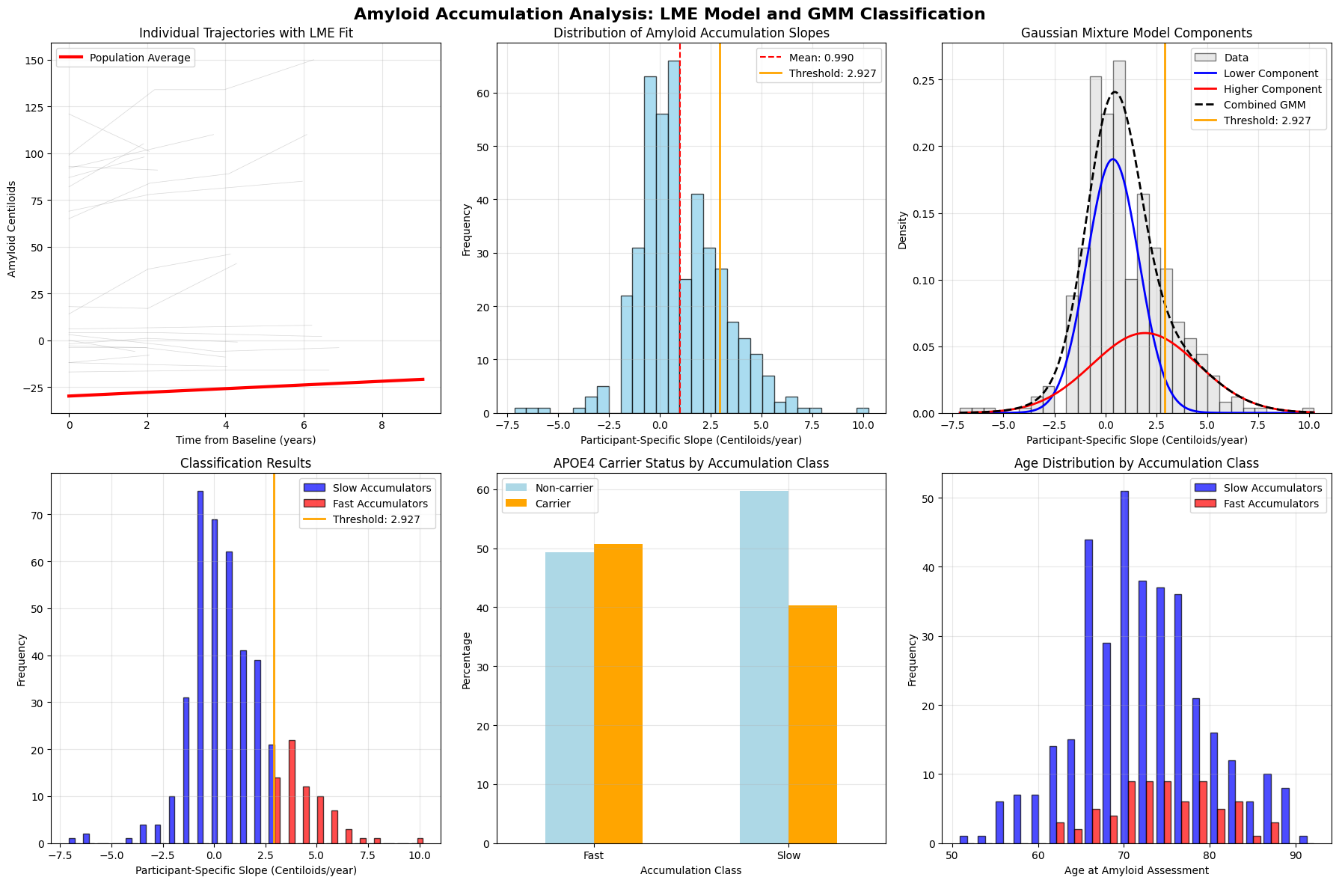

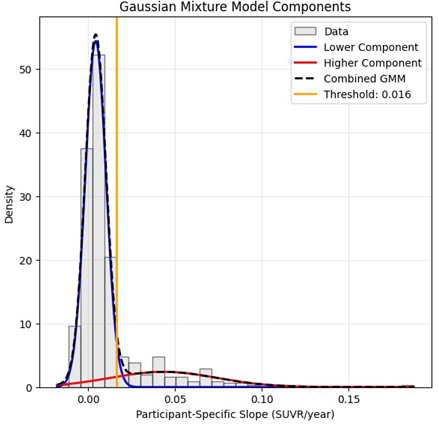


1. **Supplementary Figure 3. Correlation Matrix of Candidate Predictors**
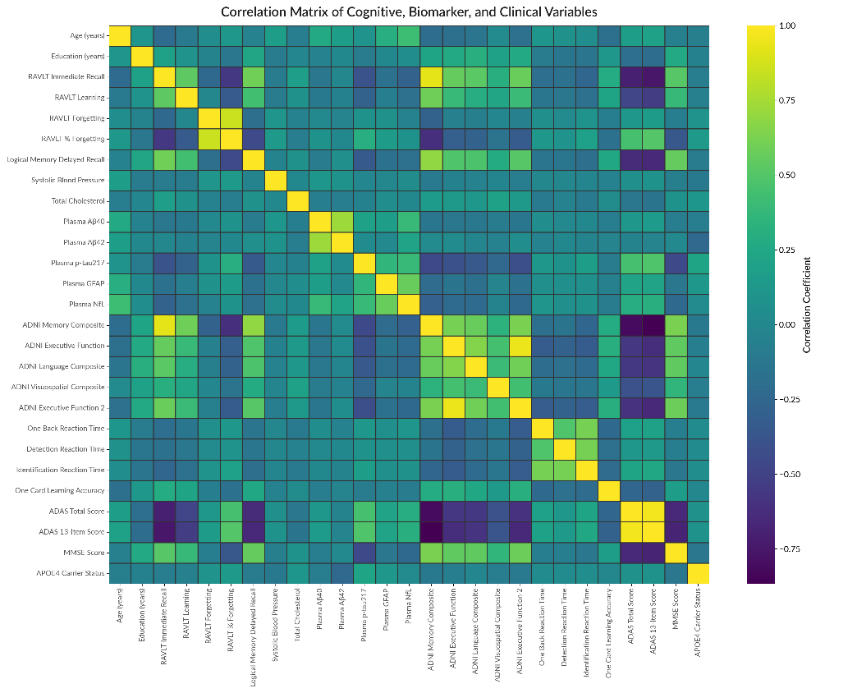

2. **Supplementary Figure 4. ROC curves for predicting accumulation patterns in amyloid-negative individuals**


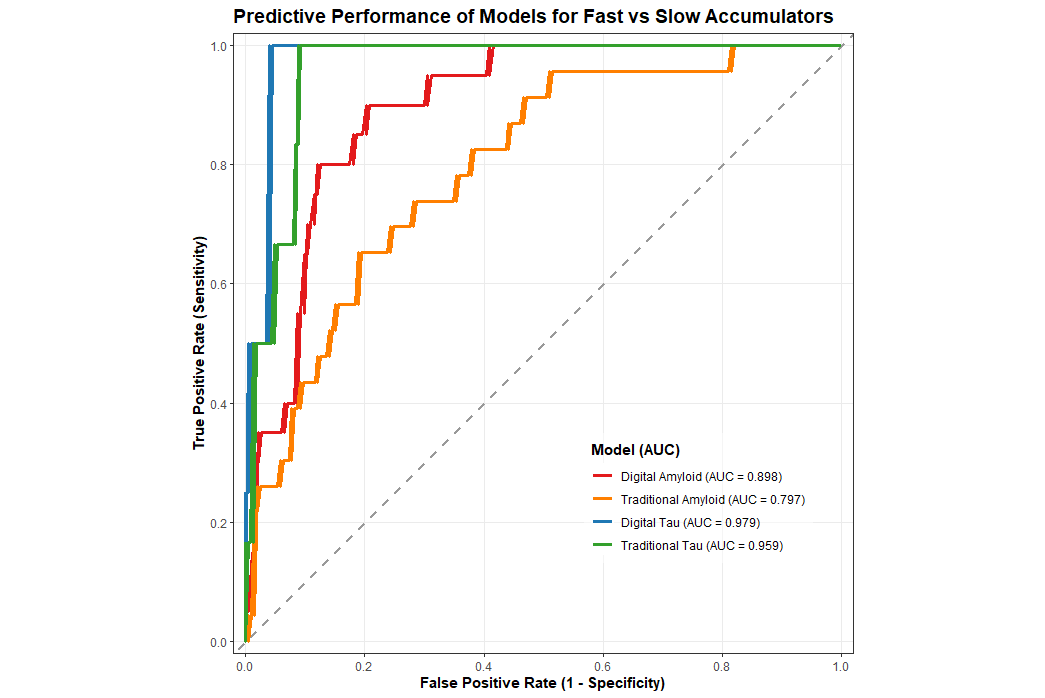

Supplement: Supplementary file 1 — Supporting Information [file DAD2-18-e70281-s001.docx]
